# Enhanced Skin Delivery of Therapeutic Peptides Using Spicule-Based Topical Delivery Systems

**DOI:** 10.3390/pharmaceutics13122119

**Published:** 2021-12-08

**Authors:** Chi Zhang, Jiwen Duan, Yongxiang Huang, Ming Chen

**Affiliations:** 1 Department of Marine Biological Science & Technology, College of Ocean & Earth Sciences, Xiamen University, Xiamen 361102, China; zhangchi@stu.xmu.edu.cn (C.Z.); duanjw@stu.xmu.edu.cn (J.D.); 2 State Key Laboratory of Marine Environmental Science, College of Ocean & Earth Sciences, Xiamen University, Xiamen 361102, China; yongxianghuang@xmu.edu.cn; 3 State-Province Joint Engineering Laboratory of Marine Bioproducts and Technology, Xiamen University, Xiamen 361102, China; 4 Shenzhen Research Institute of Xiamen University, Shenzhen 518000, China; 5 Pingtan Research Institute of Xiamen University, Pingtan 350400, China

**Keywords:** therapeutic peptides, transdermal delivery, dermal delivery, spicules, insulin, cyclosporine A

## Abstract

This study reports two therapeutic peptides, insulin (INS, as a hydrophilic model peptide) and cyclosporine A (CysA, as a hydrophobic one), that can be administrated through a transdermal or dermal route by using spicule-based topical delivery systems in vitro and in vivo. We obtained a series of spicules with different shapes and sizes from five kinds of marine sponges and found a good correlation between the skin permeability enhancement induced by these spicules and their aspect ratio *L/D*. In the case of INS, Sponge *Haliclona* sp. spicules (SHS) dramatically increased the transdermal flux of INS (457.0 ± 32.3 ng/cm^2^/h) compared to its passive penetration (5.0 ± 2.2 ng/cm^2^/h) in vitro. Further, SHS treatment slowly and gradually reduced blood glucose to 13.1 ± 6.3% of the initial level in 8 h, while subcutaneous injection resulted in a rapid blood glucose reduction to 15.9 ± 1.4% of the initial level in 4 h, followed by a rise back to 75.1 ± 24.0% of the initial level in 8 h. In the case of CysA, SHS in combination with ethosomes (SpEt) significantly (*p* < 0.05) increased the accumulation of CysA in viable epidermis compared to other groups. Further, SpEt reduced the epidermis thickness by 41.5 ± 9.4% in 7 days, which was significantly more effective than all other groups. Spicule-based topical delivery systems offer promising strategies for delivering therapeutic peptides via a transdermal or dermal route.

## 1. Introduction

Peptides are bio-polymers built up from amino acids that have been utilized as therapeutic agents by virtue of their high selectivity, sufficient efficiency, good tolerance, and affordable cost since the advent of insulin one century ago. The medical applications of therapeutic peptides are thriving and have emerged nowadays against almost all diseases, ranging from cancer to even obesity [1,2,3,4,5,6]. Today, more than 60 therapeutic peptides have been approved for pharmaceutical use worldwide, with over 600 potential ones currently being evaluated in clinical trials or pre-clinical development [7,8].

Besides the intrinsic weaknesses of peptides, including poor chemical and physical stability, relatively high molecular weight, low intestinal permeability, and short plasma half-life, the application of therapeutic peptides is limited by challenges in their delivery [9,10,11,12]. So far, almost all therapeutic peptides are administrated by injection [13], with only a few oral therapeutic peptides in existence, such as cyclosporine A [14]. Alternative administration routes of therapeutic peptides are gaining increasing interest to broaden their pharmaceutical applicability. Skin delivery of therapeutic peptides offers many advantages over systemic administration (injection or oral administration), such as the evasion of the first-pass effect, controlled drug release over time, direct access to local lesions, and good patient compliance, among others [15,16]. However, the skin barrier, composed primarily of *Stratum Corneum* (SC), generally results in low skin absorption and, consequently, low bio-availability of therapeutic peptides [17]. In order to overcome the skin barrier for enhanced skin delivery of therapeutic peptides, lots of methods have been utilized, including physical methods such as iontophoresis (calcitonin [18,19], nafarelin [20], triptorelin [21], octreotide [22]), electroporation (Insulin [23], etanercept [24,25,26]), and microneedles (desmopressin [27], erythropoietin [28], melanostatin [29], rigin [29], palmitoyl pentapeptide [29]); and chemical ones, such as penetration enhancers (heparin [30,31], cyclosporine A [32], Luteinizing Hormone Releasing Hormone [33]) and nanoparticles (IFN-α [34], cyclosporine A [35]), among others. Physical methods are usually involved with the relatively high cost and uncomfortable feeling (burns or pain) to the skin. In contrast, chemical ones usually have limited effectiveness for enhanced skin delivery. In addition, long-term use of some chemical penetration enhancers can even damage the skin barrier structure, causing skin irritation and allergy. Sponge *Haliclona* sp. spicules (SHS) have recently been utilized as scattered microneedles to disrupt the skin barrier and enhance the skin penetration of a series of therapeutics and even nanoparticles [36,37,38]. SHS can physically disrupt skin in a dose-dependent manner and be retained within the skin for over 72 h, which provides a convenient, safe, and effective skin delivery strategy.

In this study, we obtained a series of spicules with different shapes and sizes from five kinds of marine sponges, including *Haliclona* sp., *Mycale phyllophila*, *Tedania anhelans*, *Tethya* sp., and *Cliona celata*, and investigated the relationship between the spicules’ morphological characteristics and their skin-penetration-enhancing effectiveness. Two therapeutic peptides were selected in this study. One is insulin (INS), a hydrophilic peptide for diabetes treatment, and another one is cyclosporine A (CysA), a hydrophobic peptide for psoriasis treatment. We further demonstrate that INS and CysA can be administrated via a transdermal or dermal route using proper spicule-based topical delivery systems in vitro and in vivo.

## 2. Materials and Methods

### 2.1. Materials

Phospholipon 90G was purchased from Lipoid (Ludwigshafen, Germany). Fluorescein isothiocyanate insulin was purchased from Zhongkechenyu Biotech (Beijing, China). Insulin was purchased from Wanbang (Xuzhou, China). Cyclosporine A was purchased from Solarbio (Beijing, China). All other chemicals were purchased from Sinopharm (Shanghai, China).

### 2.2. Preparation and Characterization of Sponge Spicules

Sponge *Haliclona* sp., *Mycale phyllophila, Tedania anhelans,* and *Tethya* sp. were obtained from cultured explants in Dongshan Bay (Fujian, China). Sponge *Cliona celata* was obtained as a gift from Jinan University (Guangdong, China.) Different sponge spicules were prepared according to a patented method (ZL201610267764.6). The morphology of spicules was photographed by scanning electron microscopy (FEI, Quanta 650 FEG, Hillsboro, OR, USA), and the size was calculated by ImageJ software (V1.8.0).

### 2.3. Preparation and Characterization of Liposomal Systems

Liposomal systems were prepared using the thin-film hydration method [39,40]. Appropriate cyclosporine A (5 mg/mL in final formulation) and lipids, phospholipon 90G (4%) with BRIJ^®^O20 (1.2%) (flexible liposomes), or phospholipon 90G (4%) only (ethosomes) were dissolved in methanol/chloroform (2:1, *v/v*) solution and then dried by a rotary evaporator and dispersed with ethanol solution (45%, *v/v*) or PBS solution (pH 7.4, 50 mM). Then, the above suspension was extruded 21 times through a polycarbonate membrane of 100 nm pore size (AVESTIN, Ottawa, ON, Canada). The morphology of vesicles was observed by TEM (FEI, Talos F200X S, Hillsboro, OR, USA). The particle size and ζ-potential of the liposomal systems were analyzed for over one week by a Dynamic light scatterer (Wyatt, NanoStar, Santa Barbara, CA, USA).

### 2.4. Skin Penetration Study In Vitro

An in vitro skin penetration study was carried out to assess the permeation behavior of therapeutic peptides induced by spicules-based delivery systems. According to previously published methods [36,37], full-thickness porcine skin was prepared by removing fatty tissue and cutting hair shafts and then punched into disks with a diameter of 36 mm. To ensure the integrity of the skin barrier, skin conductivity was measured using a multimeter (Fluke f15b, Everett, WA, USA). Skin disks with an initial skin conductivity of less than 10 μA were used for further studies.

The skin disks with SC layer upward were mounted on the Franz diffusion cells (FDC; diffusion area of 1.77 cm^2^ and receptor volume of 12 mL). For the SC peeling group, the SC layer was tape-striped 30 times using tape (Scotch^®^ Transparent Tape, Saint Paul, MN, USA). For the spicules treatment groups, the appropriate amount of spicules (2, 5, and 10 mg) was applied to the skin and massaged for 2 min. For the Dermaroller treatment group, the skin surface was rolled with a microneedle roller (Dermaroller^®^ HC902, 0.2 mm, 162 microneedles; Munich, Germany) in different directions for 2 min. Other groups were left untreated. Skin conductivity was measured again. A receptor chamber filled with PBS (pH 7.4, 0.2 M) was thermostated at 37 °C and stirred at 600 rpm. FITC-INS formulations of 200 μL with different dosages (0.05, 0.1, and 0.2 mg/mL) were occlusively applied in the donor chamber of FDC. The CysA formulation (5 mg/mL, 200 μL) was non-occlusively applied.

After the skin penetration experiment, the receptor phase of 1 mL was withdrawn from the receptor chamber of FDC. The skin surface was washed 5 times using PBS (pH 7.4, 0.2 M). The SC layer was tape-striped 10 times using tape (Scotch^®^ Transparent Tape, Saint Paul, MN, USA). Viable epidermis was separated from the dermis by a scalpel. The dermis was then cut into small pieces. The peptides deposited in skin tissue were extracted with 4 mL of methanol using a shaker (Zhichu ZQZY-88BH, Shanghai, China, 180 rpm, 12 h). The FITC-INS concentration was analyzed with a micrometer reader (Tecan Infinite 200 PRO, Männedorf, Switzerland) with an excitation of 495 nm and an emission of 525 nm. The concentration of CysA was analyzed using HPLC (Thermo, UltiMate3000, Shanghai, China) with a C18 chromatographic column (Agilent, ZORBAX, Beijing, China). The mobile phase was acetonitrile (80%) and 5% phosphoric acid solution (20%), with a rate of 1.0 mL/min. The method was validated for linearity, accuracy, and precision. The linear range of FITC-INS during the measurements was from 0.01 to 10 μg/mL (R^2^ = 0.9999). The linear range of CysA during the measurements was from 10 to 1000 μg/mL (R^2^ = 0.9996).

### 2.5. Confocal Microscopy Study

The skin samples were collected and cryo-sectioned with a freezing microtome (Leica, CM1860 UV, Wetzlar, Germany). All sections were observed and photographed using a confocal microscope (Carl Zeiss, LSM780NLO, Jena, Germany) with an excitation of 490 nm and an emission of 530 nm.

### 2.6. Development of Diabetic Animal Models and Psoriatic Animal Models

Wistar rats (200~250 g, male) and BALB/c mice (6~8 weeks old, female) were purchased from Vitalriver Laboratory Animal Technology Co., Ltd. (Beijing, China) and raised in controlled conditions in the Xiamen University Laboratory Animal Center. All animal experiments were carried out in accordance with the requirements of the Institutional Animal Care and Use Committee (Xiamen University, China). The ethics approval number is XMULAC20170258.

Wistar rats were used to develop the diabetic rat model. Wistar rats were intraperitoneally injected with streptozotocin (Solarbio Biotechnology Co., Ltd., Beijing, China) at a dose of 65 mg/kg. After 72 h, the blood glucose level (BGL) was measured by glucometer (One Touch, New Brunswick, NJ, USA). Rats with BGL higher than 13.8 mM/L were used in further experiments.

BALB/c mice were used to develop the psoriatic model. BALB/c mice were anesthetized with glutaraldehyde (4%, 0.1 mL/20 g). The back hair of the mice was shaved. Imiquimod (IMQ) cream of 31.25 mg (5%; Mingxin Pharmaceuticals, Chengdu, China) was topically and non-occlusively applied to the skin for 7 consecutive days to develop imiquimod-induced psoriatic mice.

### 2.7. Treatment of Diabetes In Vivo

Diabetic rats were anesthetized with choral hydrate (10%, 0.3 mL/100 g) after fasting overnight, and their back hair was shaved with an electric razor. A donor chamber (1.77 cm^2^) was fixed on the back of the rat using mucilage gumwater (Ailete 408, Shenzhen, China). Blood (50 μL) was then collected by lateral tail vein laceration to determine the initial blood glucose level and insulin concentration. For the SHS group, 10 mg SHS was applied on the back skin of the diabetic rats and massaged with an electric massage applicator for 2 min. Then, 200 μL of insulin solution with different concentrations (20 and 100 U/mL) was applied to the donor chamber. For the injection group, 100 μL of insulin (10 U/mL) was injected into the rats’ abdomens. For the control group, no treatment was applied. Blood of 50 μL was collected at 1, 2, 4, 6, 8, 12, 16, and 24 h. BGL was measured by glucometer (One Touch, New Brunswick, NJ, USA) immediately. Insulin concentrations were measured by ELISA kit (Sigma Aldrich, Shanghai, China) according to the manufacturer’s instructions.

### 2.8. Treatment of Psoriasis In Vivo

Psoriasis mice were treated with CysA using different delivery systems. For the control group, the mice were observed for 7 consecutive days without any treatment. For the subcutaneous injection group, 100 μL of CysA (3 mg/mL) hydroethanolic solution (45%, *w/w*) was injected into a fixed point at the back skin of the mouse for 7 consecutive days. For the hydroethanolic solution group, 200 μL of CysA (5 mg/mL) hydroethanolic solution (45%, *w/w*) was evenly applied on the lesion area of mice back skin over 7 consecutive days. For the SHS in combination with ethosomes (SpEt) group, after 10 mg SHS treatment, 200 μL of ethosomes solution containing CysA (5 mg/mL) was evenly applied on the lesion area of mice back skin for 7 consecutive days.

Accumulation of CysA in relative organs and tissues was detected according to the published method [41]. Briefly, blood (500 μL) was collected from the mice orbit and mixed with 4 mL ether. CysA in the blood sample was extracted by strongly shaking the mixture for 5 min. The mixture was centrifuged at 4000 rpm for 10 min, and the ether layer was collected and evaporated. Then, 100 μL of methanol was added to dissolve the CysA. Mice skin, liver, and kidney samples were collected and weighed. The CysA in these tissues was extracted with 2 mL of methanol by shaking (180 rpm, 12 h). The concentration of CysA in blood and tissues was determined by HPLC. In addition, the mice skin in the treatment area and the non-treated area was cryo-sectioned and stained with hematoxylin and eosin. The epidermal thickness of each skin sample was measured at three random sites with ImageJ software (V1.8.0).

### 2.9. Statistical Analysis

All data were presented as mean ± standard deviation (SD). Statistical analysis was performed using one-way analysis of variance, followed by Student’s *t*-test. *p* < 0.05 is considered to be significant.

## 3. Results

### 3.1. Increased Skin Permeability by Topical Application of Spicules

**Figure 1:**
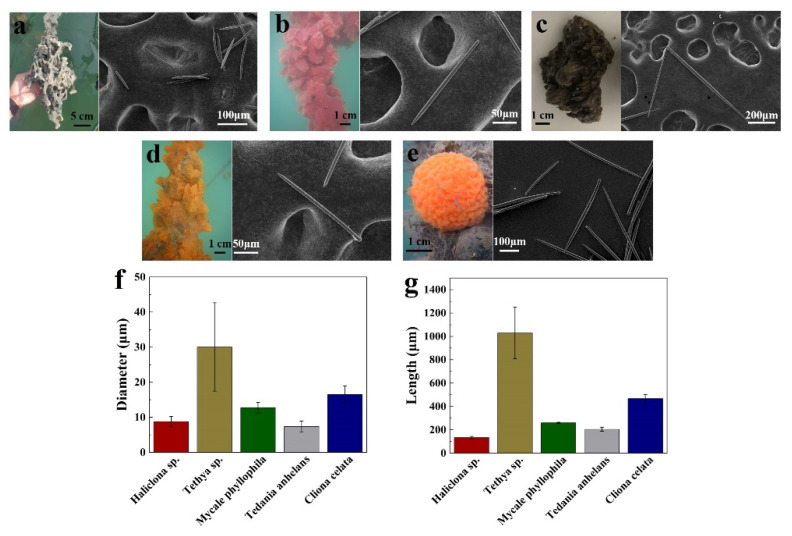
Visualization and characterization of different sponges and their spicules. (**a**) Sponge *Haliclona* sp. and its spicules. (**b**) Sponge *Mycale phyllophila* and its spicules. (**c**) Sponge *Cliona celata* and its spicules. (**d**) Sponge *Tedania anhelans* and its spicules. (**e**) Sponge *Tethya* sp. and its spicules. (**f**) Diameters of different sponge spicules. (**g**) Lengths of different sponge spicules.

A series of spicules was obtained from different sponges, including *Haliclona* sp., *Mycale phyllophila*, *Tedania anhelans*, *Tethya* sp., and *Cliona celata* and then visualized with SEM. Sponge *Haliclona* sp. spicules (SHS, Figure 1a) are spindle-shaped oxeas. Sponge *Mycale phyllophila* spicules (Figure 1b), sponge *Cliona celata* sp. spicules (Figure 1c), and sponge *Tedania anhelans* spicules (Figure 1d) are unicuspidate subtylostyle spicules. Sponge *Tethya* sp. spicules (Figure 1e) are composed of two types of spicules: strongyloxea and spheroxyaster. In addition, all these spicules were characterized by their diameter (Figure 1f) and length (Figure 1g).

**Figure 2:**
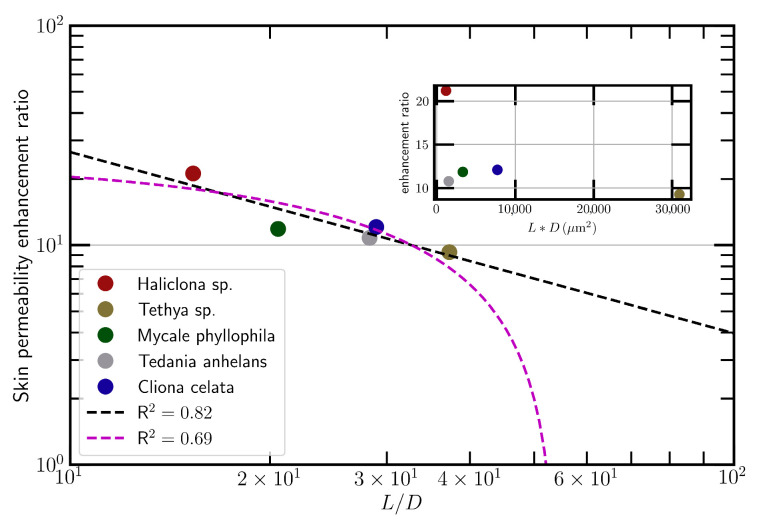
The correlation between the skin permeability enhancement ratio (*ER*) and the aspect ratio of spicules (*L/D*). Both linear and power-law fittings are shown as purple and black dash lines, with an R^2^ of 0.69 and 0.82, respectively.

The topical application of different spicules at the same dosage (1.33 mg/cm^2^) resulted in the enhancement of skin conductivity by varying degrees. We found a good correlation between the skin permeability enhancement ratio (*ER*) induced by spicules and the aspect ratio of spicules (*L/D*) (Figure 2, log_10_(*ER*) = 2.25 − 0.83log_10_(*L/D*), R^2^ = 0.82). Moreover, SHS led to the highest skin permeability enhancement compared to other spicules (Figure 2, red spot). Consequently, SHS was selected to be used for transdermal or dermal delivery of therapeutic peptides.

### 3.2. Enhanced Transdermal Delivery of Insulin In Vitro

**Figure 3:**
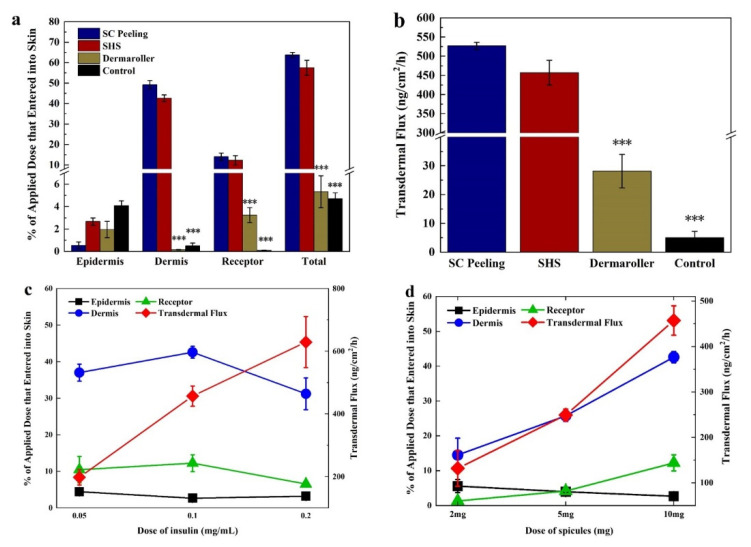
SHS enhanced transdermal delivery of insulin in vitro. (**a**) Skin absorption of FITC-INS (0.1 mg/mL) in different skin layers using different treatment strategies. (**b**) Transdermal flux of FITC-INS (0.1 mg/mL) induced by different treatment strategies. (**c**) Skin absorption and transdermal flux of FITC-INS as a function of peptides dosage. (**d**) Skin absorption and transdermal flux of FITC-INS as a function of SHS dosage. *** represents *p* < 0.001.

We assessed the transdermal delivery of insulin using different strategies in vitro, including SHS treatment, Dermaroller@200 μm (DR), SC peeling treatment, and passive penetration. FITC-labeled insulin (FITC-INS) was used for easy measurement and observation. By its passive penetration, most of the FITC-INS was accumulated in the epidermis layer (SC and viable epidermis) (Figure 3a, black bar). While the deposition of FITC-INS in deep skin layers (dermis and receptor) was only 0.6 ± 0.3% by its passive penetration, the SHS treatment resulted in 54.8 ± 3.9% of FITC-INS being deposited in deep skin layers, which was close (*p* = 0.059) to the increment induced by the SC peeling treatment (63.2 ± 1.1%) and significantly higher than that (3.4 ± 0.7%) induced by DR treatment (Figure 3a). Further, SHS treatment significantly increased the transdermal flux of FITC-INS (457.0 ± 32.3 ng/cm^2^/h) compared to DR (28.1 ± 5.8 ng/cm^2^/h) and passive penetration (5.0 ± 2.2 ng/cm^2^/h) (Figure 3b). In addition, we found that skin transdermal delivery of INS was both INS-dose-dependent (Figure 3c) and SHS-dose-dependent (Figure 3d).

**Figure 4:**
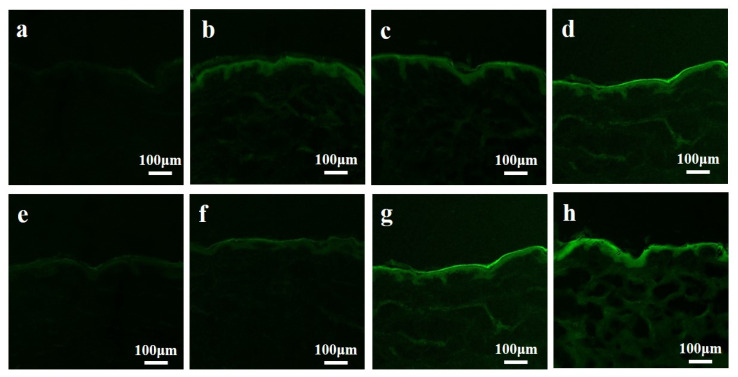
Confocal image of skin penetration of FITC-INS from (**a**) the control group; (**b**) 1.13 mg/cm^2^ spicules treatment with 0.1 mg/mL FITC-INS; (**c**) 2.82 mg/cm^2^ spicules treatment with 0.1 mg/mL FITC-INS; (**d**) 5.65 mg/cm^2^ spicules treatment with 0.1 mg/mL FITC-INS; (**e**) the control group; (**f**) 5.65 mg/cm^2^ spicules treatment with 0.05 mg/mL FITC-INS; (**g**) 5.65 mg/cm^2^ spicules treatment with 0.1 mg/mL FITC-INS; (**h**) 5.65 mg/cm^2^ spicules treatment with 0.2 mg/mL FITC-INS.

Moreover, confocal microscopy was used to evaluate skin penetration and distribution of FITC-INS after using different SHS and FITC-INS dosages in vitro (Figure 4). The amount of fluorescent staining in skin slices was dependent on both the SHS and FITC-INS doses used. The topical application of SHS (5.65 mg/cm^2^) (Figure 4d,h) showed a moderately strong FITC-INS staining from the SC to the deep dermis layer, confirming the quantitative experimental results mentioned above.

### 3.3. Transdermal Delivery of Insulin In Vivo

**Figure 5:**
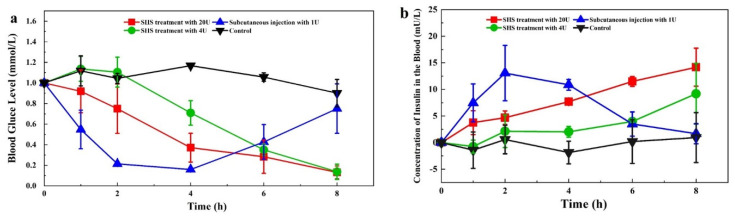
The blood glucose level (BGL) profiles and plasma insulin level (PIL) profiles induced by different treatment strategies in vivo. (**a**) BGL profiles in diabetic rats. (**b**) PIL profiles in diabetic rats.

We next investigated the blood glucose level (BGL) profiles in diabetic rats with insulin administration by subcutaneous injection and SHS treatment (Figure 5a). For the control group, the BGL of diabetic-induced rats was maintained at a high level, with small fluctuations over time. Subcutaneous injection of 1 U INS resulted in a rapid BGL reduction to 21.4 ± 2.0% of the initial level in 2 h; it slowly reached the lowest point (15.9 ± 1.4% of initial BGL) in 4 h. Afterwards, the BGL gradually rose back to 75.1 ± 24.0% of the initial level at 8 h. In contrast, the SHS treatment (5.65 mg/cm^2^), with 20 U INS, provided a slow and consistent decline to 13.1 ± 6.3% of the initial level at 8 h. The SHS treatment (5.65 mg/cm^2^) with 4 U INS showed a similar BGL profile, with a slower decline curve. On the other hand, we found that the plasma insulin level (PIL) profiles, induced by the different treatments, were inversely related to the BGL profiles (Figure 5b). The AUC (area under the curve) of SHS treatment (5.65 mg/cm^2^) with 20 U INS was 66.3 mU/Lh, which is higher than the subcutaneous injection of 1 U INS (60.4 mU/Lh). The peak time (T_max_) for subcutaneous injection was 2 h, which was much faster than SHS treatment. Compared to the subcutaneous injection of 1 U INS, the transdermal relative bioavailability of SHS treatment with 4 and 20 U was 2.2% and 5.5%, respectively.

### 3.4. Enhanced Dermal Delivery of CysA In Vitro

**Figure 6:**
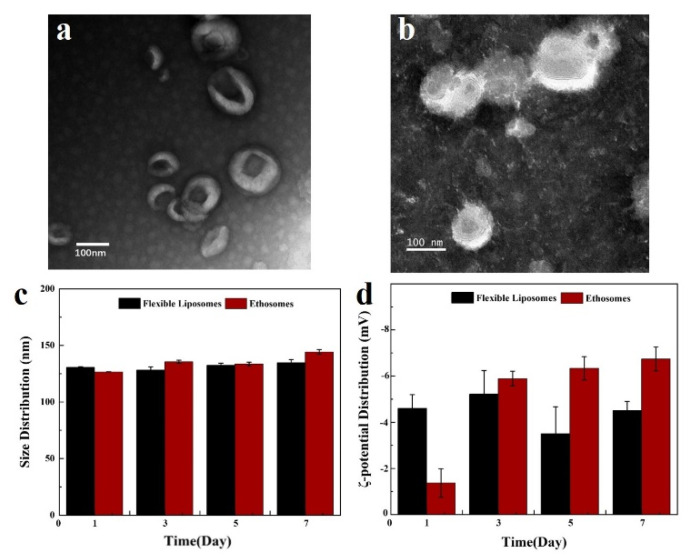
Visualization and characterization of ethosomes and flexible liposomes. (**a**) TEM image of ethosomes. (**b**) TEM image of flexible liposomes. (**c**) Particle size profiles of ethosomes and flexible liposomes over 7 days at 4 °C. (**d**) ζ-potential profiles of ethosomes and flexible liposomes over 7 days at 4 °C.

**Figure 7:**
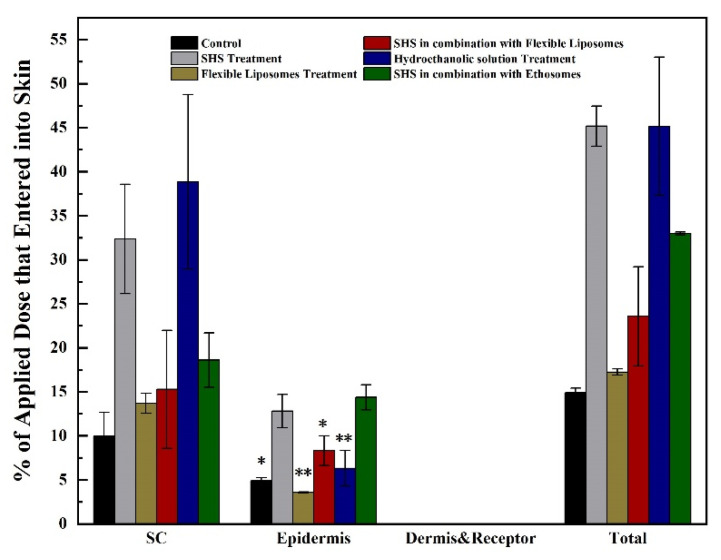
SHS in combination with ethosomes (SpEt) significantly increased the accumulation of CysA in viable epidermis in vitro. * represents *p* < 0.05, ** represents *p* < 0.01.

We also investigated the dermal delivery of CysA using different penetration-enhancing strategies in vitro. Due to its hydrophobic nature, CysA is dissolved by hydroethanolic solution (45%, *w/w*) or encapsulated into nano-carriers such as liposomes or ethosomes for its topical application. Visualization and stability results of two nano-carriers are shown in Figure 6. For the dermal delivery of CysA in all test formulations, more than half of the CysA delivered into the skin was accumulated in the SC layer; the rest of it was accumulated in viable epidermis, and no peptide deposition in the dermis was observed (Figure 7). SHS in combination with ethosomes (SpEt) increased the deposition of CysA in viable epidermis (14.4 ± 1.4%) compared to the SHS group (12.8 ± 1.9%, non-significantly, *p* = 0.45) and all other groups (significantly, *p* < 0.05)). In addition, while SHS treatment significantly (*p* < 0.05) increased the deposition of CysA in viable epidermis (12.8 ± 1.9%) compared to the control group, there was no significant difference (*p* = 0.06) between SHS in combination with flexible liposomes and control.

### 3.5. Dermal Delivery of CysA In Vivo

**Figure 8:**
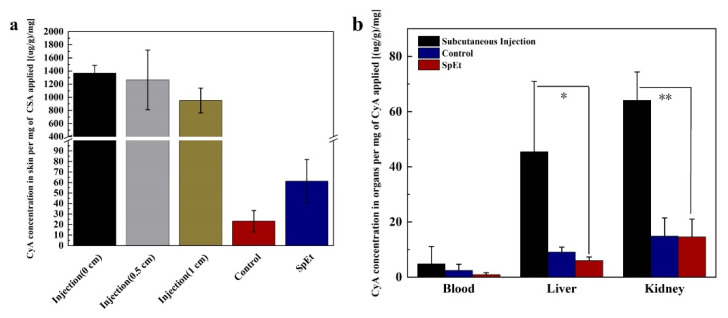
The combined use of ethosomes and SHS(SpEt) enhanced CysA skin penetration and reduced its systemic deposition in vivo. (**a**) The skin deposition of CysA induced by different treatments over 7 days in vivo. (**b**) Biodistribution of CysA induced by different treatments over 7 days in vivo. * represents *p* < 0.05, ** represents *p* < 0.01.

We next investigated the skin deposition and tissue distribution of CysA in psoriasis mice after its subcutaneous injection and topical application in vivo. Compared to its topical application, the subcutaneous injection of CysA (7 times, from day 1 to day 7) led to high drug deposition in the skin around the injection site, and the accumulation amount was inversely proportional to the distance to the injection center (Figure 8a). However, subcutaneous injection of CysA resulted in much higher drug accumulation in mice liver (45.42 ± 25.53 (μg/g)/mg, *p* = 0.0164) and kidney (64.07 ± 10.25 (μg/g)/mg, *p* = 0.0065) compared to its topical application (Figure 8b), which could further increase the risk of potential side effects of CysA treatment. The topical application of SHS in combination with ethosomes (SpEt) significantly (*p* = 0.0457) increased the skin absorption (61.26 ± 20.62 (μg/g)/mg) of CysA compared to its passive penetration (23.18 ± 10.23 (μg/g)/mg) from ethanol solution (45%, *w/w*) (Figure 8a). In addition, the accumulation of CysA in the liver and kidney, induced by SpEt, was very limited and similar (*p* > 0.05) to those induced by its passive penetration group (Figure 8b).

**Figure 9:**
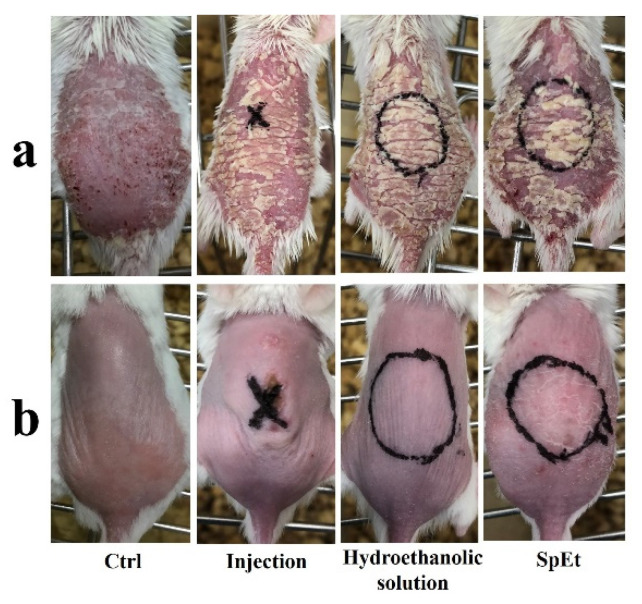
Psoriatic symptoms in mice back skin. (**a**) Dorsal skin of mice induced by IMQ for 7 consecutive days. (**b**) Dorsal skin of mice after different treatments.

**Figure 10:**
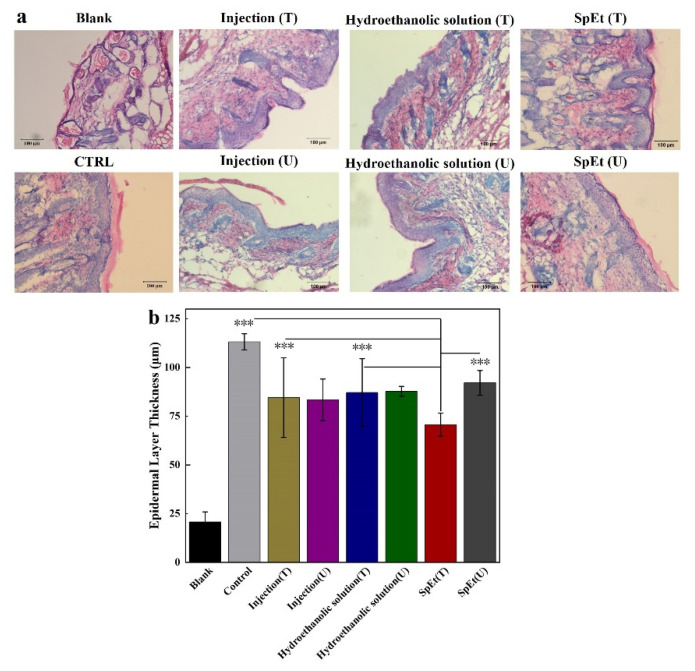
Treatment of psoriasis in vivo. (**a**) Representative H&E staining of cross-sectional slices from the dorsal skin after different treatments on day 14. (**b**) Epidermal thickness of the dorsal skin after different treatments on day 14. T represents the treatment area; U represents the untreated area. *** represents *p* < 0.001. Each bar represents the mean ± SD (*n* = 12). BLANK represents normal (non-psoriatic) mouse skin.

We further assessed the effect of CysA on the psoriasis mice by its subcutaneous injection and topical application in vivo (Figure 9). After 7 days of treatment using different strategies, the mice skin in the treatment area was cryo-sectioned and stained with hematoxylin and eosin (Figure 10a). The mice epidermis thickness before and after treatment was determined and analyzed (Figure 10b). The psoriasis mice epidermis thickness was significantly (*p* < 0.001) reduced by SpEt treatment (70.7 ± 5.9 μm) compared to the lesion thickness without SpEt treatment (92.2 ± 6.4 μm), the one around or away from the subcutaneous injection (84.6 ± 20.4 μm and 83.5 ± 10.6 μm, respectively), the one with or without passive penetration from ethanol solution (87.2 ± 17.3 μm and 87.8 ± 2.5 μm, respectively), and control (113.2 μm ± 4.1 μm), suggesting that dermal delivery of CysA using SpEt is an effective and promising strategy for the psoriasis treatment.

## 4. Discussion

The advantages of peptides as therapeutics can be offset by the challenges in their delivery. The injection of therapeutic peptides is usually less appealing for chronic diseases treatments, especially ones that need to be administered frequently. The oral delivery of therapeutic peptides is severely hampered by their high polarity as well as large molecular weight and the existence of gastrointestinal tract (GIT) enzymes [42,43]. The skin delivery of therapeutic peptides is usually limited by the SC barrier despite the fact that it can avoid both GIT degradation and first-pass metabolism with better patient compliance [16]. SHS has been utilized as a novel kind of microneedle to create plenty of micro/nano-channels across the SC layer and, consequently, enhance the skin delivery of therapeutics or even nanoparticles [37,38]. Thus, the enhancement of skin permeability induced by spicules should be positively correlated with the amount of micro/nano-channels per unit area and channel pore size. The amount of micro/nano-channels should be proportional to the number of spicules per unit weight and subsequently be inversely proportional to the volume of a single spicule (LD^2^). On the other hand, the channels induced by spicules are circular gaps (G) between skin and spicules. Considering the channels’ pore size is ¼π((D + G)^2^ − D^2^), the pore size should be proportional to the spicules’ diameter (D), considering that G (size of nm) is much smaller than D (size of μm). Finally, the enhancement of skin permeability induced by spicules should be inversely proportional to the product of the spicules’ length and diameter (*LD* product). However, experimental data suggest a bad correlation between them (the inserted figure in Figure 2). In contrast, we found a better correlation between the skin permeability enhancement induced by spicules and their aspect ratio *L/D* (Figure 2). Both linear and power-law fittings are considered via a least-square fit algorithm. They provide a slope of −0.46 and a scaling exponent −0.83, respectively, with R^2^ being 0.69 and 0.82. Visually, the power-law fitting provides a better description of the experiment data. It also provides a reasonable asymptotic value zero when *L/D* approaches infinity while the linear fitting is unbounded. Note that the power–law relation is often found in a complex system where the so-called similarity emerges spontaneously. However, due to the limited number of experimental cases, the power–law relation should be verified in the future with more cases, e.g., *L/D* > 40 or *L/D* < 10. Further, it should be pointed out that although porcine skin is widely accepted as a surrogate for human skin, it is a critical variable to make predictions on the behaviors of spicules on skin when using different skin models. More studies need to be carried out in the future, with more spicules and in ex-vivo human skin, to confirm the correlation found in this study. In addition, this correlation is very preliminary. The exact physical significance of the good correlation between *ER* and *L/D* could be further investigated in the future.

INS has a narrow therapeutic index as a peptide medicine [44]. Further, the different durations of action in mealtime and basal therapy as well as the high inter- and intra-patient variability of INS complicate its delivery [45]. Compared to injection (1 U of insulin injected, AUC = 60.4 mU/Lh), the transdermal route (SHS treatment with 20 U INS) can achieve a higher level of INS absorption (AUC = 66.3 mU/Lh) within 8 h. Compared to subcutaneous injection of 1 U INS, the transdermal relative bioavailability of SHS treatment with 4 and 20 U in 8 h was 2.2% and 5.5%, respectively. However, the absorption of insulin by the transdermal route was sustained over time (Figure 5b). Transdermal relative bioavailability of SHS treatment could be increased for a longer duration. The micro-channels created by the Dermaroller gradually close up in 20 min [36,37] due to the self-contraction of elastic skin, resulting in limited INS permeation time and, therefore, quite low INS bio-availability. In contrast, SHS can be inserted into the skin and retained over 48 h, which ensures the INS has enough time to permeate into circulation and, subsequently, improve bio-availability. Further, the transdermal INS delivery induced by SHS was both INS-dose-dependent (Figure 3c) and SHS-dose-dependent (Figure 3d), which could meet the complexity of the INS delivery.

CysA is a hydrophobic therapeutic peptide [46] that has been utilized clinically for the treatment of numerous inflammatory and autoimmune diseases [47]. In the treatment of dermatological diseases such as psoriasis and alopecia areata, among others, dermal delivery of CysA with low transdermal absorption is often preferred to its systemic administration in order to reduce the potential systemic toxicity of the drug [48]. However, dermal delivery of CysA is usually hindered by its large molecular weight (1202 Da), hydrophobic nature (log P_octanol/water_ = 2.9), as well as the incrassated SC in psoriasis [49,50,51,52]. We topically applied IMQ, a potent immune activator, to trigger psoriasis-like inflammation, according to a previous study [53]. After continuous application of IMQ cream over 7 days, the mic skin exhibited symptoms of erythema, scaling, and thickening (Figure 9a), which was basically similar to human psoriasis. For enhanced CysA skin absorption, a hydroethanolic solution (45%, *w/w*) was used as an optimal vehicle compared to other ones, such as pure ethanol, ethyl oleate, transcutol, and isopropyl myristate, among others, according to previous research [41]. However, we noticed that the control (non-treated) mouse seemed to recover from psoriasis lesions better than the treated mice did (Figure 10a). Actually, psoriasis developed by Imiquimod in mice is self-limited, which is also in accord with published research [49,54]. Psoriasis scales gradually heal and fall off. Thus, we evaluated the severity of psoriasis and the therapeutic effectiveness of topical formulations by measuring the epidermal thickness in mice instead of the appearance of psoriasis lesions. We speculate that this phenomenon could result from the long-time topical application of hydroethanolic solution (45%) on treated mice. Long-time application of high concentrations of alcohol on skin could result in the dehydration and dryness of lesion skin. On the other hand, we found that SHS in combination with flexible liposomes can dramatically enhance the skin delivery of hydrophilic biomacromolecules such as hyaluronic acid (250 KDa) [37]. Consequently, we combined SHS with ethosomes (liposomes in a hydroethanolic solution) to develop a topical delivery system of CysA (SpEt) for maximizing its deposition in viable epidermis. In the future, the commercial formulations of ethosomes containing CysA could be prepared as a lyophilized powder. Before usage, the powder could be re-hydrated with a corresponding solvent (hydroethanolic solution). From the results (Figure 4c,d), the ethosomes were stable for over one week, which could be quite enough for its topical application in its liquid form.

For the in-vivo experiment, we compared the untreated area skin thickness (U) with the treated one (T) in the same mouse. Further, we also compared the epidermis thickness in the treated area (T) with the control (the epidermis thickness in untreated mice). We noticed two phenomena: (1) the epidermis thickness of the control was significantly thicker than the untreated area skin thickness (U) in the treated mice; (2) there was no significant difference between the untreated area skin thickness (U) and the treated one (T) in the same mouse except for the SpEt group. We speculate that the CysA deposited in the epidermis of the treated skin area (T) could diffuse into the epidermis of the untreated skin area (U). Compared to all other groups, SpEt could further enhance the deposition of CysA in the epidermis, resulting in the significantly decreased epidermis thickness of psoriasis lesions.

## 5. Conclusions

This study shows that the skin permeability enhancement induced by spicules is inversely proportional to their aspect ratio (*L/D*). Topical applications of SHS can significantly enhance the transdermal delivery of INS both in vitro and in vivo. The combined use of SHS and ethosomes (SpEt) can significantly increase the accumulation of CysA in viable epidermis in vitro and effectively reduce the epidermis thickness of psoriasis mice, with minimal systemic side effects in vivo. Spicule-based topical delivery systems offer promising strategies for delivering therapeutic peptides via the transdermal or dermal route.

## 6. Patent

Ming Chen reports an authorized patent (US 10555896B2).

## Data Availability

The data presented in this study are available on request from the corresponding author.
